# Parents ASSIST: Acceptability and Feasibility of a Video-Based Educational Series for Sexuality-Inclusive Communication between Parents and Gay, Bisexual, and Queer Sons

**DOI:** 10.3390/ijerph19010379

**Published:** 2021-12-30

**Authors:** Dalmacio D. Flores, Kate Hennessy, Andre Rosario, Jamie Chung, Sarah Wood, Trace Kershaw, Antonia Villarruel, Jose Bauermeister

**Affiliations:** 1 Department of Family and Community Health, University of Pennsylvania, Philadelphia, PA 19104, USA; khenn@nursing.upenn.edu (K.H.); arosario@nursing.upenn.edu (A.R.); jamiechung224@gmail.com (J.C.); amvillar@nursing.upenn.edu (A.V.); bjose@nursing.upenn.edu (J.B.); 2 Craig A. Dalsimer Division of Adolescent Medicine, Children’s Hospital of Philadelphia, Philadelphia, PA 19104, USA; woodsa@chop.edu; 3 Department of Social and Behavioral Sciences, Yale School of Public Health, New Haven, CT 06510, USA; trace.kershaw@yale.edu

**Keywords:** HIV prevention, online intervention, MSM

## Abstract

Young men who have sex with men (YMSM) between the ages of 13 and 24 are a key population for HIV prevention. The parents of gay, bisexual, and queer (GBQ) adolescent males and the sex communication they have with their sons has yet to be explored as an HIV prevention intervention. We developed an online video series called Parents ASSIST (Advancing Supportive and Sexuality-Inclusive Sex Talks) to educate parents about sexual health topics pertinent to GBQ males. We pilot tested the series with a sample of 54 parents of GBQ males using a single-group post-test design. Participants viewed the videos and completed a survey measuring the acceptability and feasibility of an online video series to educate parents. Most of the parents (70.4%) believed that the videos would make parents more likely to initiate talking about sex with their sons. The results suggest that online videos are an acceptable way for parents to learn about GBQ sexual health topics.

## 1. Introduction

HIV and STIs disproportionately affect gay, bisexual, and queer (GBQ) adolescent males. In 2018, GBQ adolescent males accounted for 92% of new HIV diagnoses among all 13- to 24-year-olds, and 21% of new HIV diagnoses among all gay, bisexual, and queer male age groups [1]. Racial HIV disparities remain salient within this population, with Black and Latinx GBQ adolescent males disproportionately affected by HIV diagnoses [2]. Sexual risk behaviors, including condomless intercourse, early debut, and forced sex, are also more prevalent among GBQ adolescent males compared to their heterosexual counterparts [3]. Sexual risk behaviors are also associated with negative mental health in GBQ adolescent males [4]. The hostile and non-accepting ecological structures in which GBQ youth often operate are linked to negative mental health outcomes including higher reports of depression, suicidal ideation, and emotional distress compared to their heterosexual counterparts [3,5]. For instance, 40% of GBQ adolescent males stated that they had seriously considered suicide, compared to 11% of their heterosexual counterparts [5].

Despite the negative outcomes noted, LGBTQ youth, including GBQ adolescent males, report earlier ages for self-identification and for coming-out compared to prior LGBTQ generations [6]. A 2021 Gallup poll reported an increase in the number of U.S. adults identifying as LGBTQ from 4.5% in 2017 to 5.6% in 2020 [6]. This increase may be linked to growing acceptance of same-sex relationships and sexual behaviors in society [7]. Generation Z, or those born between 1997 and 2012, includes the greatest proportion of LGBTQ-identifying individuals [6]. Among a national sample of gay and bisexual men in the U.S., Grov et al. (2018) found that a vast majority (83.6%) of those sampled reported having their first same-sex attractions at an average age of 10.4; the average age that GBQ men reported having told someone about their identity was 17.9 [8]. Earlier disclosure of sexual identity may afford more opportunities to provide LGBTQ youth with inclusive information, that is, sexuality-specific support and education. In turn, this support and education can impact GBQ adolescent males’ behavior and act as health-protective factors. Ecological determinants, including parental communication, can function as health-protective factors, given the proximal relationship parents have with GBQ youth.

Parents are in an opportune position to serve as sexuality information and support providers for their LGBTQ children [9]. Parents are able to communicate with children before sexual debut [10] and educate and support LGBTQ teens as they become aware of their attractions, behaviors, and identities [11]. As shown in our previous work, GBQ males in the Southern U.S. have expressed wanting to learn about sexual health from their parents rather than from other sources such as health-care providers, teachers, or the internet [12]. Underscoring their unique same-sex attractions and experiences that are different from cisgender sexual diverse females (lesbian, queer, etc.) and gender diverse youth (transgender, gender nonbinary, etc.), GBQ males contend with incessant homophobia and navigate oppressive and homophobic systems that ignore their emerging sexual health education needs [13,14]. Family relationships have been shown to impact the sexual behaviors of LGBT youth through close relationships and through conversations about being cautious and using condoms [15].

Effective sex communication at home improves heterosexual adolescents’ self-efficacy with condom use, skills to resist pressure to have sex, readiness to initiate conversations about HIV/STI prevention before engaging in sex with partners, and use of reproductive and sexual health services [16,17,18,19,20,21]. Sex communication between mothers and adolescents in particular demonstrates a protective role resulting in safer sex behavior among adolescents [21]. Parent–child sex communication is associated with later sexual initiation, less at-risk sex behavior, and increased condom use in adolescence [22,23]. While past research offers a list of evidence-based interventions for sex communication between heterosexual adolescents and their parents [16], there is a dearth of interventions for sex communication involving parents with non-heterosexual children, including parents of GBQ adolescent males [11,24,25]. To our knowledge, despite 40 years of PCSC research, there is no published intervention focused on parents with LGBTQ youth. Recent literature reviews have similarly reported the lack of GBQ-specific intervention work that aims to address the sexual health outcomes of this subpopulation [9,21,26]. Further, GBQ youth face unrelenting standards of hegemonic masculinity as they come of age [12] and have an array of sexual health concerns that differ markedly from those of their heterosexual counterparts, making modifications to current interventions for heterosexual adolescents particularly challenging for GBQ youth.

Parental acceptance and sexuality-specific support are linked to feelings of affirmation and fewer identity struggles for LGBTQ individuals [27], while family rejection or lack of support is associated with negative sexual and mental health outcomes [28,29]. Morris et al. (2020) found that among GBQ adolescent males from three major urban U.S. cities who were living with parents or guardians, those whose mothers had provided accepting spaces and positive reactions to disclosures of sexual identity were less likely to engage in sexual risk behaviors [30]. Family support was also protective in terms of reducing the prevalence of reporting four or more sexual partners in the previous 12 months [4].

Parents of LGBTQ youth are not only well-positioned to provide support to their children on health-related topics, but they are also eager to do so. Rose et al. (2014) found that 90% of parents reported that health communication was moderately to very important to them, highlighting feelings of connectedness as a major benefit [31]. Many parents view sex communication with their GBQ sons as part of their parental responsibility even when their sons resist or are uncomfortable [32]. This drive to communicate about engaging in safer sex demonstrates the further positive impact that parents can have on sexual health outcomes for GBQ sons when parents are given relevant information and tools.

Previous work has found that parents describe sex communication as interactive discussions, while GBQ sons describe their conversations as vague; the direction they receive about safer sex is often too broad or lacks information specific to their sexual identities [15]. Parents give the same advice to their heterosexual children as they do to their non-heterosexual children [33], indicating discomfort or a lack of knowledge around sex topics relevant to GBQ adolescent males [15,34,35]. During sex communication, African American parents and GBQ sons report that HIV and sexual orientation are the most discussed topics [31], which may be driven by fear, lack of specific sexual health knowledge, or discomfort with other topics such as sexual activity. LaSala (2015) also found from a diverse racial/ethnic sample that GBQ adolescent males report that they are waiting for their parents to initiate sex communication, while at the same time, parents are expecting their sons to be the ones to initiate conversation [15]. This misalignment in expectations creates a gap in communication and prevents successful initiation of sexuality-focused parent–child sex communication.

These reported inadequacies signal opportunities to improve sex communication between parents and their GBQ sons. Other parent-reported barriers to sex communication with GBQ sons have included a lack of accurate knowledge, effective peer-parental role models to emulate, and confidence to initiate and sustain conversations about sex [9,24]. Parental discomfort and feelings of ineptitude around discussing sex can impede discernible research findings on the effectiveness of parent–child sex communication for GBQ sons [36]. Equipping parents of GBQ adolescent males with the tools and knowledge to initiate and sustain sex communication will allow for a clearer picture of the protective role that parent–child sex communication plays in health outcomes for GBQ sons as well as the effectiveness of sex communication interventions for this population.

The Parents ASSIST (Advancing Supportive and Sexuality-Inclusive Sex Talks) video series was designed to be an online resource for parents to (1) learn about the pertinent sexual health topics germane to GBQ youths’ sexuality concerns and (2) receive communication skills for initiating and sustaining sex discussions with GBQ sons. The Theory of Planned Behavior (TPB) and Social Cognitive Theory (SCT), which have been used as frameworks to influence parents’ intentions to communicate about sexual health in previous research [21,37], serve as the theoretical backbones of our video content. TPB rests on the idea that an individual’s attitudes, subjective norms, and perceived behavioral control influence that individual’s intention to perform a behavior [38,39], including the intention to communicate about inclusive sexual health with a GBQ son.

To address parental attitudes, or positive or negative beliefs about a behavior, the videos prompt parents to examine their attitudes toward more inclusive sex talks and to reflect on personal experiences with sex communication with their own parents. The videos aim to support parents’ positive attitudes about health issues pertinent to GBQ sons’ identities. To address subjective norms or perceived social influences to engage or not engage in a behavior, the videos reference other parents’ normative beliefs about sex communication from larger, heteronormative ecological systems around the home [12]. The main characters of the video series include parents who share their similar concerns and situations. Regarding perceived behavioral control, or one’s self-efficacy to perform a behavior, the videos prompt parents to examine their capacity to initiate and sustain inclusive talks. Social Cognitive Theory (SCT) posits that by observing others perform a behavior, including inclusive sex communication, individuals learn to engage in that behavior [40]. The videos capitalize on observational learning by modeling inclusive sex communication, normalizing parental hesitance to address sex with GBQ sons, and encouraging frequent attempts to engage GBQ sons. Our primary aims for this Parents ASSIST pilot study were to (1) determine the feasibility of enrolling parents of GBQ youth into an online study about targeted sexual health discussions at home, (2) measure the acceptability of animated videos as a means to learn about GBQ-specific topics and communication skills, and (3) collect parental feedback about individual video content and the overall video series.

## 2. Methods

### 2.1. Intervention Description

The Parents ASSIST project is an online series of 13 animated videos. For this pilot study, the videos were presented on a website that also described the study aims, listed the study team members, and offered additional online resources for sexual health information. The central video in this series, “Answering Questions”, runs for 12 min, and the remaining 12 videos range from 4 to 6 min in length. The cast of animated characters represents people from different cultural backgrounds through dress, gender, ethnicity, and age, as well as through situations that reflect common and shared parenting concerns across diverse populations. Previous research has found that culturally tailored eHealth videos could be used to increase awareness of HIV/STI prevention methods since the viewers may identify with the characters, which lessens their resistance to the health message [41,42,43]. In addition, Bandura (2001) recommended that characteristics of the models should be similar to the viewers’ to increase the impact of educational modeling [40]. Aside from the racially and ethnically diverse animated characters, the Parents ASSIST videos feature at least 48 sexual health topics and a multitude of family contexts to ensure its appeal to our target participants.

### 2.2. Intervention Development Procedures

We worked alongside community members to develop the video series [13]. The initial framework for the videos was established using recollections from GBQ sons who reported salient issues in parent–child sex communication [12,44]. Using that foundation, advisory groups were formed. First, a Parent Advisory Board was assembled. It was comprised of parents of GBQ cisgender males, school educators who identified as gay, and health-care professionals with LGBTQ health experience. We explored their thoughts about existing resources, determined their intervention priorities, and identified any logistical concerns around intervention work with the target population. The Parent Advisory Board members suggested that a video-based intervention would appeal to other parents and be a convenient way to learn about sexual health topics and communication strategies. Over the course of a year, they met four times to select video topics that appealed to parents and to evaluate how relatable and realistic the video scripts appeared. Ultimately, the Parent Advisory Board guided screenplay development for the videos, provided content expertise, and helped ensure that the topics recommended by GBQ youth were included throughout the development of the intervention [13].

Second, an Interdisciplinary Group of Nurses and Health Educators (IGNHE) was convened to ensure that the videos presented clinically reliable information. Members included pediatricians, public health researchers, an AIDS-certified nurse, and a sexual health educator. They offered feedback on the scripts being developed by the parents. The combined feedback from groups of health-care professionals and parents contributed to multiple stages of developing the videos, website, and study procedures. During subsequent meetings, advisory group members provided their suggestions on the quality of the animated characters’ voices, the conversations between central characters, and the overall appeal of the videos. This cyclical engagement that lasted for over a year starting in 2018 between advisory group members and program staff enabled the iterative co-development and refinement of a theoretically-driven video series and website cognizant of the unique parenting challenges that our target audience faces. The iterative process of organizing these groups and integrating their feedback into different stages of developing the intervention has been detailed elsewhere [13].

### 2.3. Participant Recruitment

Once the videos and website were developed, we recruited an online sample of English-speaking parents whose GBQ sons were between the ages of 12 and 24 years to participate in online surveys. Participants were recruited nationally through advertisements on Facebook and through word-of-mouth from community organizations local to Philadelphia. Advertisements directed at children to recruit their parents were approved by our Institutional Review Board. Eligibility criteria included having a male son between the ages of 12 and 24 years old who identified as gay, bisexual, or queer. As an incentive, participants were offered a $50 Amazon gift card.

### 2.4. Study Procedures and Measures

Eligible participants first completed an online consent form. Then, they watched 3 videos from the series. Afterwards, a post-test survey was conducted. The survey also allowed participants to write free-text comments. The survey measured acceptability and feasibility along the following dimensions: relatability, likeability, utility, recommendability, realistic quality, and potential impact (Table 1). After survey data collection was completed, 6 parents (about 10% of the sample) who provided the highest and lowest survey ratings were contacted for a chance to view the remaining Parents ASSIST videos they had not seen and to participate in a one-on-one interview.

## 3. Findings

### 3.1. Demographic Findings

Table 2 provides demographic details of the study sample. The parents who participated in our study (N = 54) had a mean age of 44.5 (SD = 5.56) and included 30 mothers (55.6%) and 24 fathers (44.4%). Most participants identified as Caucasian/White (*n* = 44, 81.5%) followed by African American/Black (*n* = 5, 9.3%). Other participants were Biracial (*n* = 3, 5.6%), Asian/Pacific Islander (*n* = 1, 1.9%), or Native American/Alaskan (*n* = 1, 1.9%). The majority of participants identified as Non-Hispanic/Non-Latino (*n* = 47, 87%).

The participants’ sons were an average age of 16.98 (SD = 2.57), with a majority in high school (*n* = 30, 55.6%), while some were in college/vocational school (*n* = 11, 20.4%) or middle school (*n* = 8, 14.8%). Most participants reported that their sons self-identify as gay (*n* = 48, 88.9%) or bisexual (*n* = 5, 9.3%) while one had a queer-identifying son (1.9%). According to the sample, over one-third of sons had come out more than 2 years ago (*n* = 20, 37%), and many of them had come out within the past 1–2 years (*n* = 16, 29.6%). Some participants reported their sons coming out more recently—about 18.5% of them (*n* = 10) between 6 and 12 months ago, and another 14.8% (*n* = 8) within the past 6 months.

### 3.2. Feasibility

Pilot study results show that parents engaged with the Parents ASSIST videos from start to finish. Participants took an average of 41 min to complete watching the required three videos (“Answering Questions”, 1 topic-based video, and 1 communication-based video). The time reflects pausing videos and providing responses to pop-up questions we programmed to capture in-the-moment feedback. The total average time from participants signing consent to the final survey question was 71 min with 52 of the 54 parents completing the online survey in a single sitting.

### 3.3. Acceptability

Table 3 summarizes the survey questions related to six dimensions of acceptability. Regarding relatability, 27.8% (*n* = 15) of participants agreed that “I definitely related to most or all the characters”, while 44.4% (*n* = 24) could relate to “many of the characters”. 20.4% (*n* = 11) of participants described the series as “extremely interesting”, 61.1% (*n* = 33) as “very interesting”, and 14.8% (*n* = 8) as “moderately interesting”. On a question where participants indicated how realistic the videos appeared to them, 75.9% (*n* = 41) indicated “definitely yes”, while 24.1% (*n* = 13) indicated “maybe”, and none indicated “definitely no”.

In measuring likeability, 75.9% (*n* = 41) of participants indicated “definitely yes” when rating how much they liked what the main characters were saying. In asking participants if they thought other parents might like to see the videos, 27.8% (*n* = 15) said “most or all would”, while the majority indicated “many would” (*n* = 33, 61.1%). When asked how much participants liked the video series, 51.9% indicated the highest rating, “very much” (*n* = 28).

Our survey also measured knowledge utility. When asked if participants thought that watching the videos would help increase parents’ knowledge about their sons’ health and sexuality questions, the majority indicated “definitely yes” (*n* = 47, 87%). When asked if participants thought that the videos could make it more likely that other parents would seek further information about LGBTQ health and sexuality, the majority indicated “definitely yes” (N = 37, 68.5%). Finally, in asking the participants if the videos added to their learning about LGBTQ health and sexuality issues, two-thirds answered, “definitely yes” (*n* = 36, 66.7%). Gauging how much new information the participants felt they learned, 59.3% (*n* = 32) noted they learned “a great deal”, while 35.2% (*n* = 19) reported learning “some” new information.

In terms of recommendability, 90.7% (*n* = 49) of participants indicated that they would recommend the animated videos to other parents of GBQ adolescent males, while 5 parents indicated “no”. Relatedly, 85.2% (*n* = 46) of participants would recommend watching the videos with their sons. When asked if they would share the videos with their friends, 79.6% (*n* = 43) indicated “definitely yes”. With regards to whether the videos seemed realistic, 64.8% (*n* = 35) reported “definitely yes”. In response to “Would you describe their situations as realistic”, 75.9% (*n* = 41) answered, “definitely yes”.

The survey asked questions about the video series’ perceived potential to impact communication. When asked if the videos could help parents initiate and sustain sex and health discussions with their GBQ sons, the majority of participants (*n* = 38, 70.4%) answered, “definitely yes”. When asked if the videos would prompt parents to initiate inclusive conversations about health and sexuality with their sons, answers were consistent; 70.4% (*n* = 38) of participants answered, “definitely yes”. When asked if the videos could make it more likely that other parents do the same, 74.1% (*n* = 40) answered, “definitely yes”. We also asked if participants thought the videos could help parents sustain inclusive conversations about health and sexuality with their sons and 68.5% (*n* = 37) answered, “definitely yes”. Finally, we asked participants if they thought that the videos could help parents learn how to handle conversations about health and sexuality with adolescent sons and broach issues/questions centered on same-sex attractions or behaviors. The majority (*n* = 43, 79.6%) indicated “definitely yes”.

Parents rated the videos using a 6-point scale (1—very poor, 2—poor, 3—fair, 4—good, 5—very good, 6—excellent). They also supplied qualitative feedback via pop-up boxes after each video was viewed in its entirety. The ratings and qualitative feedback for each individual video are listed in Table 4.

### 3.4. Overall Impressions of Parents ASSIST

Six parents who provided the highest and lowest survey ratings were contacted to view the remaining Parents ASSIST videos they had not seen and to participate in one-on-one interviews. All 6 parents agreed to participate. Interviewed via Zoom, most of the parents (*n* = 4) were recruited online when they saw the study flyers on social media, while 2 of them were referred to the study through snowball sampling. After being granted access to all 13 of the videos and being asked to watch the video series in its entirety, participants were asked about their general impressions of Parents ASSIST, the website, and its format and content. The 30–60 min post-survey interviews were transcribed and thematically organized according to positive and negative impressions with corresponding codes assigned to each unique comment. These positive and negative impressions from parents are listed in Table 5.

## 4. Discussion

There is a growing cohort of U.S. parents who are accepting and supportive of their GBQ sons with same-sex attractions, behaviors, or identities. Given the continued exclusion of this subpopulation’s sexual health concerns in school-based sex education programs, parents may be an untapped HIV/STI sexual health resource. The Parents ASSIST videos are a parent–child sex communication intervention iteratively co-developed with extensive community input with key stakeholders and expert groups. To our knowledge, this study is one of the first to test an inclusive sex communication intervention that addresses a longstanding health disparity issue associated with this youth population. Our study established the feasibility of enrolling parents of GBQ youth into an online study about targeted sexual health discussions at home and to measure the acceptability of animated videos as a means to learn about GBQ-specific topics and communication skills.

Preliminary findings from this pilot study indicate that parents of GBQ males have a high likelihood of participating in online educational interventions that use animation technology. Among parents recruited, 44% were fathers, which indicates high interest on the topic not just among mothers who have traditionally been the parent who engages in these conversations [24]. More than half of participants had GBQ sons in high school and, despite most of the sons having come out to their parents more than 2 years ago, parents reported high interest and use for the intervention, which reflects the lack of resources around inclusive sexual health parenting [31]. Most parents viewed the three required videos in a single sitting and responded to our pop-up questions. Additionally, a third of the sample indicated interest in participating in future Parents ASSIST research after completing the surveys. Other parents indicated willingness to be contacted for other similar studies after they completed the consent forms. These promising findings suggest that a series of animated videos about topics pertinent to sexuality, health, and inclusive communication skills is a feasible way to reach parents who would like to be sexual health resources for their GBQ sons.

The acceptability of the intervention was high among the study participants, with parents overwhelmingly endorsing the animated videos across six domains. A majority of the parents could relate to the characters and stories depicted, and most found the videos interesting and realistic. An overwhelming number of participants liked how the main characters responded to the communication situations presented and envisioned their friends as liking these videos as well. From the surveys, parents indicated that the videos increased their knowledge about sexual health issues they can communicate with their sons. Likewise, there was general agreement among participants that if made available, they would recommend the animated series to other parents of GBQ males, as they could see how it would result in initiating and sustaining sexual health discussions. Taken together, the high acceptability ratings across the six domains indicate positive parental attitudes to the intervention medium. In particular, the repeated endorsements of the videos to friends or other parents of GBQ males signal how Parents ASSIST may be a mechanism to influence subjective norms to engage in sexuality-specific sexual heath communication. Given the parental fear that talking about sex with youth may encourage adolescent sexual experimentation [45,46], participants’ endorsements of the videos to other parents may indicate a readiness to change subjective norms or larger normative beliefs within the ecological settings that these parent–child dyads navigate. Finally, with parents attesting to an increase in their knowledge level about GBQ-specific sexual health information and a high likelihood of initiating and sustaining inclusive discussions with their GBQ sons, participants’ behavioral control or self-efficacy to engage in inclusive talks appears to have similarly increased.

Several limitations have to be noted. First, the pilot survey employed a self-selected group of parents who were recruited mainly through online groups formed around supporting LGBTQ adolescents. While parental acceptance of a GBQ child does not necessarily translate to health communication proficiency, parents in these online groups may be primed to favorably view resources focused on the needs of this youth population. Correspondingly, the views of parents who may be unaccepting of a son’s disclosure as GBQ may not be represented in our sample. Additionally, the lack of a randomized comparison group in our study design prohibits establishing intervention efficacy. Similarly, the lack of a pre-test does not allow us to examine changes in theoretical constructs at this time. As such, we are currently limited in our ability to claim Parents ASSIST’s capacity to educate parents and encourage inclusive sexual health communication. As a next step, we will be conducting a randomized controlled trial (RCT) to assess the videos’ efficacy across multiple time points. Finally, a majority of our sample was White and non-Latinx, which also limits the generalizability of our findings.

Content-wise, we note the lack of representation of single-parent households in our videos. While almost half of our participants were fathers, the omission of single-parent households in the videos was pointed out as contributing to an idealistic representation of family discussions around inclusive sexual health. Similarly, while the focus of this study was on parents addressing the sexual health needs of cisgender GBQ males, our exclusion of gender diverse youth and cisgender lesbian, bisexual, and queer females’ parallel concerns was noted by participants as a shortcoming. Our future work will include more types of family structures beyond the nuclear family and will represent other members of the LGBTQ community in the videos and health discussions. Finally, we will solicit the feedback of the GBQ youth themselves whose parents will participate in the RCT. Differences in youth and parent PCSC reports have been noted in prior studies [47], and we anticipate that GBQ youth may similarly offer a different perspective on the intervention’s efficacy in the home.

To conclude, Parents ASSIST was designed to be an online resource for parents to learn about topics germane to the sexual health of GBQ youth and to provide communication skills centered on inclusive parent–child sex communication. Findings from the study support animated videos as a feasible way of learning communication skills and sexual health for parents of GBQ males. The quality of the feedback from parents indicates an unmet need for sexuality-focused family resources tailored to the unique considerations of emerging adolescent males with same-sex attractions, behaviors, and identities. The implications of our findings may be extended to intervention work involving families with LGBTQ children.

## Figures and Tables

**Table 1:** Project Dimensions and Sample Survey Questions.

Dimension	Survey Question
**Relatability**	Q1. Could you relate to the characters? Q2. How interesting were the videos you watched? Q3. Would you describe their situations as realistic?
**Likeability**	Q4. Did you like what the main characters were saying? Q5. Do you think other parents might like to see the videos? Q6. How much did you like the video series you just watched?
**Utility**	Q7. Do you think that watching the videos could help increase parents’ knowledge about their sons’ health and sexuality questions? Q8. Do you think the videos you watched could make it more likely that other parents will seek further information about LGBTQ health and sexuality? Q9. Did the videos add to your learning about LGBTQ health and sexuality issues? Q10. How much new information did you learn as a result of the videos you just watched?
**Recommendability**	Q11. Would you recommend the animated videos to other parents of gay, bisexual, or queer adolescent males? Q12. Would you recommend watching these videos with your soon? Q13. Would you share the videos with your friends?
**Realistic Quality**	Q14. Were the stories realistic? Q15. Would you describe their situations as realistic?
**Potential to Impact Communication**	Q16. Do you think that watching the videos could help parents initiate and sustain sex and health discussions with their gay, bisexual, or queer sons? Q17. Do you think the videos you watched could make it more likely that other parents will want to initiate and sustain inclusive parent–child conversations about health and sexuality? Q18. Do you think that after watching the videos, parents will be more likely to initiative inclusive conversations about health and sexuality with their sons? Q19. Do you think that after watching the videos, parents will be more likely to sustain inclusive conversations about health and sexuality with their sons? Q20. Do you think the videos could help parents learn how to handle conversations about health and sexuality with adolescent sons and broach issues/questions centered on same-sex attractions or behaviors?

**Table 2:** Demographic Profile.

	Frequency (*n* = 54)	Percentage
**Parent’s Age**		
32–40	11	20.6
41–50	35	65
53–60	6	11.3
Not specified	2	3.7
**Sex**		
Male	24	44.4
Female	30	55.6
**Race**		
Caucasian/White	44	81.5
African American	5	9.3
Asian/Pacific Islander	1	1.9
Native American/Alaskan	1	1.9
Biracial	3	5.6
**Ethnicity**		
Hispanic/Latino	7	13
Non-Hispanic/Non-Latino	47	87
**U.S. Region**		
Northeast	16	29.8
South	13	24.3
Midwest	9	16.8
West	16	29.7
**Son’s Age**		
14–16	26	48.1
17–19	17	31.5
20–24	11	20.5
**Son’s Grade**		
8th grade	8	14.8
9th grade	8	14.8
10th grade	6	11.1
11th grade	7	13
12th grade	9	16.7
College/vocational/trade school	11	20.4
Finished college/vocational/trade school	3	5.6
**Son’s Sexual Orientation**		
Gay	48	88.9
Bisexual	5	9.3
Queer	1	1.9
**Son’s Gender**		
Cisgender male	54	100
**Time Known Son is GBQ**		
1–2 months	3	5.6
2–6 months	5	9.3
6–12 months	10	18.5
1–2 years	16	29.6
2–4 years	10	18.5
More than 4 years	10	18.5

**Table 3:** Acceptability Measure Results.

Domains *	Responses N (%)				
**Relatability**					
Q1.	I definitely related to most or all the characters	I could relate to many of the characters	I could relate to some of the characters	I don’t know if I could relate to any of the characters	No, I could not relate to any of the characters
	15 (27.8)	24 (44.4)	14 (25.9)	1 (1.9)	0 (0)
Q2.	Extremely interesting	Very interesting	Moderately interesting	Slightly interesting	Not at all interesting
	11 (20.4)	33 (61.1)	8 (14.8)	2 (3.7)	0 (0)
Q3.	Definitely Yes	Maybe	Definitely No		
	41 (75.9)	13 (24.1)	0 (0)		
**Likeability**					
Q4.	Definitely Yes	Maybe	Definitely No		
	41 (75.9)	13 (24.1)	0 (0)		
Q5.	Most or all would	Many would	I don’t know	A few would	None would
	15 (27.8)	33 (61.1)	4 (7.4)	2 (3.7)	0 (0)
Q6.	Very much	Somewhat	Neutral	Not much	Not at all
	28 (51.9)	22 (40.7)	1 (1.9)	3 (5.6)	0 (0)
**Utility**					
Q7.	Definitely Yes	Maybe	Definitely No		
	47 (87)	7 (13)	0 (0)		
Q8.	Definitely Yes	Maybe	Definitely No		
	37 (68.5)	17 (31.5)	0 (0)		
Q9.	Definitely Yes	Maybe	Definitely No		
	36 (66.7)	14 (25.9)	4 (7.4)		
Q10.	A great deal	Some	Little	None	
	32 (59.3)	19 (35.2)	2 (3.7)	1 (1.9)	
**Recommendability**					
Q11.	Yes	No			
	49 (90.7)	5 (9.3)			
Q12.	Yes	No			
	46 (85.2)	8 (14.8)			
Q13.	Definitely Yes	Maybe	Definitely No		
	43 (73.6)	10 (18.5)	1 (1.9)		
**Realistic Quality**					
Q14.	Definitely Yes	Maybe	Definitely No		
	35 (64.8)	19 (35.2)	0 (0)		
Q15.	Definitely Yes	Maybe	Definitely No		
	41 (75.9)	13 (24.1)	0 (0)		
**Potential to Impact Communication**					
Q16.	Definitely Yes	Maybe	Definitely No		
	38 (70.4)	15 (29.6)	0 (0)		
Q17.	Definitely Yes	Maybe	Definitely No		
	40 (74.1)	13 (24.1)	0 (0)		
Q18.	Definitely Yes	Maybe	Definitely No		
	38 (70.4)	16 (29.6)	0 (0)		
Q19.	Definitely Yes	Maybe	Definitely No		
	37 (68.5)	17 (31.5)	0 (0)		
Q20.	Definitely Yes	Maybe	Definitely No		
	43 (79.6)	11 (20.4)	0 (0)		

* Please refer to Table 1 for the survey questions.

**Table 4:** Ratings for Each Video and Qualitative Feedback.

Video, Mean Rating, SD, Range *1—Very Poor to 6—Excellent*	Video Descriptions	Qualitative Feedback
**Communication-Skills Focused Videos**		
**Answering Questions** 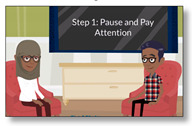 **Mean: 4.94 (0.90)** **Range: 2–6**	Depicts a conversation between a mother and son to demonstrate various verbal and nonverbal techniques that help to provide a nonjudgmental and affirming environment when answering a son’s emergent questions. Constructs addressed: Observational Learning; Self-Efficacy/Perceived Behavioral Control. (Duration: 12:14 min)	“This video was great. I learned how to manage emotions and find a proper way to communicate with my children.” -Claire, mother with a 15 y/o gay son “I like how it talked about most parents are uncomfortable talking to their kids about sex also how our facial expression can tell a lot and to answer the questions with facts take time to think about the answer.”—April, mother of 15 y/o gay child
**Overcoming Communication Barriers** 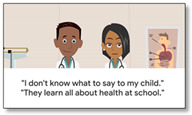 **Mean: 4.22 (0.98)** **Range: 3–6**	Nurses address misconceptions about sex communication with children to encourage parents to initiate conversations about sex with their sons. Constructs addressed: Attitudes; Subjective Norms (Duration: 5:00 min)	“It’s such a great topic! It needs to be done much sooner in life… If our culture could be taught to start talking about their bodies at an early age and age appropriately I think it would prevent a lot of issues surrounding the LQBTQ community and beyond.”—Victoria, mother with a 19 y/o gay son “It’s a great video which made me tear up. I would like to share this video to my family members and friends.”—Shawn, father of 14 y/o gay child
**Coming Out and Communication Tips** 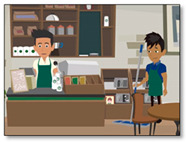 **Mean: 5.25 (0.89)** **Range: 4–6**	Presents various coming out stories. Provides guidance on affirmative responses for parents when sons come out. Constructs addressed: Attitudes; Self-Efficacy/Perceived Behavioral Control (Duration: 4:25 min)	“I really liked hearing each of the adolescents stories of how they came out. I know its incredibly hard for a teen when they come out but the video made me realize its even more complex then I originally thought. I liked the way the information was presented and it also motivated me to want to go research more online.”—Jack, father of 15 y/o gay child “It’s not a easy thing to come out for a teenager. I went through a hard time with my son when he realized he may like a boy.” —Tom, father of 15 y/o bisexual son
**Tips from Parents Like You** 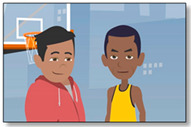 **Mean: 5.09 (0.70)** **Range: 4–6**	Offers advice on how parents can express their own views about sex in open and inclusive sex communication with their sons. Constructs addressed: Subjective Norms; Attitudes (Duration: 4:45 min)	“I was so moved by this video, and it’s very much like a story that happened in my life.”—Joanne, mother with a 15 y/o gay son “It was encouraging to talk to other parents and again see that surrounding yourself with likeminded people is helpful… I liked how all of the talk was gender and sexuality neutral because all the issues are relevant for all teens, irrespective of gender or sexual orientation.”—Robert, father of 19 y/o gay child
**You’re Normal, Kiddo!** 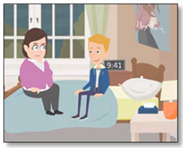 **Mean: 5.00 (0.82)** **Range: 4–6**	Normalizes LGBTQ identities and introduces various sexuality and gender-specific terms. Models parental reassurance of GBQ sons and highlights stigma experienced due to societal heteronormativity. Constructs addressed: Observational Learning; Attitudes (Duration: 5:54 min)	“I had never thought body language was important while talking and I agree with the video—it does make a big difference and is significant. The video made me think about the tone and facial expressions I should be using. I also liked the part that dealt with answering my son’s questions with facts that he will understand.”—Jack, father with a 15 y/o gay son “The scroll of terms is a reminder how much there is to learn, and how much we are not taught about sexual orientation and gender identity… I appreciate that the doctor character talked about learning from and along with his children.”—Cynthia, mother of 24 y/o gay child
**When we say “Be safe”…** 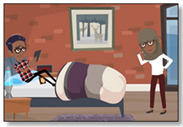 **Mean: 4.60 (0.70)** **Range: 4–6**	Emphasizes importance of ongoing sex communication that is detailed instead of general instructions about safety. Constructs addressed: Attitudes; Self-Efficacy/Perceived Behavioral Control (Duration: 6:20 min)	“I thought it presented a specific scenario that a parent could use as an example for a conversation about being safe. I like the “fail” examples also. Using humor can be a way to lessen the anxiety that might surround the issue.”—Michelle, mother with a 16 y/o queer-identifying child “It’s kind of embarrassing for me to talk with my kids about “safety” since I need to mention a lot of details. They might feel more awkward than me. This video solves the problem perfectly.”—Sheena, mother of 14 y/o gay child
**Setting Rules and the Teenager’s Developing Brain** 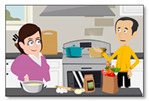 **Mean: 5.00 (0.89)** **Range: 4–6**	Models parental rule-setting that aligns with adolescent brain development. Constructs addressed: Intentions; Observational Learning (Duration: 6:15 min)	“It’s very useful.”—Franklin, father of 20 y/o gay child “The video regarding setting rules and teenagers developing brain did not seem to flow so easy for me. I also didn’t feel like that would be normal conversation between a father and a son.”—Amy, mother of 22 y/o gay child
**Information-Focused Videos**		
**Before Doing the Deed** 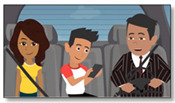 **Mean: 4.55 (0.82)** **Range: 4–6**	Suggests ways parents can provide sex education and gauge their sons’ readiness to have sex as well as their understanding of consent. Constructs addressed: Observational Learning; Subjective Norms (Duration 5:11: min)	“It’s neat how the parents took a very casual approach while talking about abstinence. Not being preachy and being realistic were also two important approaches discussed in the video that I approved of.”—Brandon, father of 17 y/o gay child “The parent-parent conversation was good. Being surrounded by others who support your view of talking about sex with your kids has to help.”—Robert, father with 19 y/o gay son
**Reassuring Conversations** 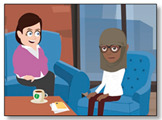 **Mean: 5.00 (0.63)** **Range: 4–6**	Describes potential ways parents can show support to sons who are facing discrimination at school, from extended family members, or in other social spaces. Constructs addressed: Subjective Norms; Observational Learning (Duration: 4:35 min)	“Great video. I wouldn’t change anything.” —Amy, mother of 22 y/o gay child “After watching I took away a few of the points such as making it clear that I can be relied upon and showing my love during our conversations.” —Brandon, father with a 17 y/o gay son
**Demystifying HIV Testing** 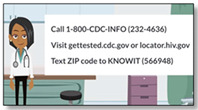 **Mean: 5.38 (0.52)** **Range: 5–6**	Provides information on HIV testing, including LGBTQ-friendly testing. Models ways parents can bolster their sons’ autonomy in navigating sexual health care structures. Constructs addressed: Attitudes; Intentions (Duration: 4:53)	“I think one of the most important things discussed was getting rid of the stigma around HIV and testing. The information was clear, straight to the point, and easy to understand.”—Jack, father with a 15 y/o gay son “This is a much harder topic to discuss with our kids. This video is really good. I do think that being part of the LGBT community, my son probably hears more on this topic that straight, cisgender boys, which is really good. Again, my favorite part was the example conversation the mom had with her son.”—Jennifer, mother of 18 y/o bisexual child
**Adolescents, Technology and Communication** 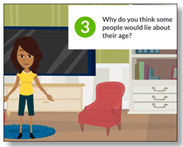 **Mean: 4.90 (0.88)** **Range: 4** **–** **6**	Suggests questions to gauge sons’ understanding of safety concerns with sexting and online dating/hook-ups and provides opportunities to offer tips for navigating these situations. Constructs addressed: Attitudes; Self-Efficacy/Perceived Behavioral Control (Duration 4:42 min)	“I feel exactly the same as the parents in this video. It’s hard to talk about safe sex since you need to let kids know what is unsafe sex.”—Jeremy, father with a 14 y/o gay son “It’s great to have communication about difficult subjects with our teens/young adults, but hard for them to know our desire to help them have safe relationships and to have an exit plan, boundaries, etc. Older dates can sound exciting albeit risky to many teens and young adults.”—Dori, mother of 22 y/o gay child
**HIV and HPV Prevention** 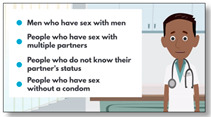 **Mean: 5.60 (0.52)** **Range: 5** **–** **6**	Describes PrEP and the HVP vaccine and CDC guidelines based on age and sexual activity. Constructs addressed: Attitudes; Intentions (Duration 5:19 min)	“Very informative and well-summarized with the most up-to-date medical information. Especially appreciate the PrEP information and I think it may be something not all parents are aware of. My son brought it to my attention.”—Paula, mother with a 21 y/o gay son “I think it was well done on how PrEP is needed for those who are gay. Safe sex is better than being unprotected. That is what I told my son.”—Derek, father of 14 y/o gay child
***Abuse and Victimization*** 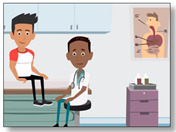 **Mean: 4.33 (0.50)** **Range: 4–5**	Demonstrates ways to discuss consent and sexual assault with sons. Offers options for reporting abuse. Constructs addressed: Self-Efficacy/Perceived Behavioral Control; Observational Learning (Duration 5:05 min)	“Good advice on listening without prying or opining. Good that there are contacts for supportive organizations.”—Sarah, mother with a 20 y/o gay son “Safety is a hard topic to talk with child since you need to tell them what activities are not safe in details. This video offers a better choice.”—Shawn, father of 14 y/o gay child

**Table 5:** Parental Feedback on the Parents ASSIST video series (*n* = 6).

Theme	Code and Description
Positive Impressions	*Step-by-step instructions* (*n* = 15) *. Parents appreciated the methodical way each video broke down a communication process or provided basic information about a topic first before building up to more complex issues. The numerous “sample scripts” parents can use in their everyday lives were regarded as one of the tangible benefits to watching both sets of videos.
	*Short video duration for self-paced viewing and schedule flexibility* (*n* = 8). With 12 of the 13 videos running under 5 min viewing time, parents felt they could watch the videos at their own pace based on their level of knowledge (beginner vs. advanced level).They could also anticipate seeing the entire series at different times depending on their schedule due to the unstructured time commitment.
	*Normalized parental struggles with non-judgmental approach* (*n* = 8). Many parents appreciated how the videos recognized the overwhelming nature of discussing sensitive sexual health matters with GBQ sons. The reassuring tone throughout the videos that did not judge parents for not having already initiated talks or have a comprehensive understanding of LGBTQ health created a friendly atmosphere to learn.
	*Diversity of characters and spectrum of family experiences and conundrums* (*n* = 9). Viewers positively regarded the diverse range of animated characters along with the different situations depicted in the videos as seemingly realistic. Parents found the series inclusive of different ethnic/racial individuals with relatable plot lines.
	*Easy website navigability with apt titles, descriptions, and resources* (*n* = 16). Noting the “clean” layout with ample white space, parents overwhelmingly had no issues exploring the Parents ASSIST website and selecting the videos that interested them based on the titles and descriptions. Parents noted the comprehensive topic-specific resources embedded at the end of each video as well as the broad online links listed on the “Resources” tab of the website.
	*Compendium of parenting tips for improved communication* (*n* = 5). Parents appreciated the extensiveness of communication tips they heard in the videos, which repeated the value of making up for lost sex talk opportunities, admitting knowledge limitations, and partnering with sons to look up LGBTQ-specific information parents might not have ready answers about.
	*Acknowledgment that GBQ sons may sometimes know more than parents* (*n* = 3). Noting that youth typically come out to parents after they reach a certain level of comfort with being GBQ, several parents appreciated the recognition that sons often possess more knowledge than their parents do about LGBTQ health issues. Videos that depict sons as informed about rudimentary issues pertaining to their own health were favorably received and evaluated as realistic.
	*Other Positive Feedback* Addressed misconceptions about GBQ sexuality and barriers to talking about sex; Showed a good range of typical family settings (e.g., in a car, playground, health clinic, school); Covered the importance of nonverbal communication such as facial expressions, pauses, and head nods; Provided reminders to fact-check what sons claim to know about certain issues; Information came from an interdisciplinary team of health experts.
Negative Impressions	*Broad focus and undefined youth age targets* (*n* = 3). Two parents commented on the vast number of topics covered throughout the series that they judged may be too advanced for parents with elementary-age GBQ sons (e.g., PrEP) or too basic for those with sons already in high school or about to go to college (e.g., what the acronym LGBTQ means). These parents suggested clearly identifying the target age range for each video to help plan for when they would be most appropriate to use.
	*Idealized or unrealistic dialogue* (*n* = 3). Two parents reported some of the dialogue they heard as too idealistic, formal, or unrealistic. According to them, youth depicted in the videos seem to speak maturely while most parents in real life would be unable to deliver some of the recommended lines as they seemed too scripted.
	*Technical glitches* (*n* = 3). Two parents reported experiencing occasional difficulty viewing the videos on their Android phones or tablets. According to them, some videos took a while to load and some of the descriptions did not line up to the appropriate video image.
	*Lack of content addressing lesbian and bisexual daughters and transgender children* (*n* = 5). A few parents noted that they would have appreciated videos that tackled gender-specific issues (e.g., use of pronouns, transgender health, etc.) or included concerns of families with lesbian or bisexual daughters.
	*Other Negative Feedback* Lack of single-parent households in the videos; Lack of guidance on sexuality health talks prior to a child’s coming out; Need for local-level resources parents can access in their communities; Overemphasis on harm reduction and risk mitigation with no mention of advising GBQ youth to completely abstain from certain behavior (e.g., sexting).

* *n* = the number of times a code was mentioned by participants.

## Data Availability

The data are not publicly available at this time due to ongoing analyses.
